# Daily morning light therapy is associated with an increase in choroidal thickness in healthy young adults

**DOI:** 10.1038/s41598-018-26635-7

**Published:** 2018-05-29

**Authors:** Scott A. Read, Emily C. Pieterse, David Alonso-Caneiro, Rebekah Bormann, Seentinie Hong, Chai-Hoon Lo, Rhiannon Richer, Atif Syed, Linda Tran

**Affiliations:** School of Optometry and Vision Science, Queensland University of Technology, Brisbane, Queensland Australia

## Abstract

Ambient light exposure is one environmental factor thought to play a role in the regulation of eye growth and refractive error development, and choroidal thickness changes have also been linked to longer term changes in eye growth. Therefore in this study we aimed to examine the influence of a 1-week period of morning light therapy upon choroidal thickness. Twenty two healthy young adult subjects had a series of macular choroidal thickness measurements collected with spectral domain optical coherence tomography before, and then following a 7-day period of increased daily light exposure. Increased light exposure was delivered through the use of commercially available light therapy glasses, worn for 30 minutes in the morning each day. A significant increase in subfoveal choroidal thickness (mean increase of +5.4 ± 10.3 µm) was found following 7-days of increased daily light exposure (p = 0.02). An increase in choroidal thickness was also observed associated with light therapy across the central 5 mm macular region. This study provides the first evidence in the human eye that daily morning light therapy results in small magnitude but statistically significant increases in choroidal thickness. These changes may have implications for our understanding of the impact of environmental factors upon eye growth.

## Introduction

Evidence from both human and animal studies suggest that the choroidal tissue that lines the posterior eye (situated between the retina and the sclera), plays an important role in the regulation of eye growth and the development of refractive errors. Studies experimentally inducing refractive errors in a range of animal models (including chick^1^ and primate^2^) indicate that predictable changes in the thickness of the choroid accompany the development of myopic and hyperopic refractive errors. These choroidal changes during refractive error development occur rapidly^3^ and are typically found to precede changes in scleral growth^4^. Stimuli that typically slow eye growth in the longer term (and result in the development of hyperopic refractive errors) are associated with a thickening of the choroid, whereas stimuli that result in the more rapid eye growth (and result in the development of myopia in the longer term) are typically associated with a thinning of the choroid^5^.

Similar to a range of ocular and systemic tissues and physiological processes, the choroid is also known in both animals^6^ and humans^7–10^ to undergo significant diurnal variations in thickness over the course of the day, and daily cycles of light exposure are thought to play a critical role in the synchronization of these circadian rhythms^6^. In both animal studies, and in a number of human studies examining choroidal thickness, the choroid is typically found to be thinnest during the day (typically reaching a minimum at midday or early afternoon), and thickest at night time^6,7,9,10^. In research with chicks, when the rate of eye growth increases or decreases during the development of experimental myopic and hyperopic refractive errors respectively, significant changes in the phase and/or amplitude of diurnal choroidal rhythms are observed. Interrupting the normal light/dark cycle is also known to disrupt diurnal choroidal rhythms and may influence eye growth, suggesting a role for circadian rhythms of the choroid in the regulation of eye growth^6^.

In humans, the presence of refractive error is also associated with alterations in choroidal thickness, with cross-sectional studies indicating that a thinner choroid is associated with the presence of myopia, and a thicker choroid with the presence of hyperopic refractive errors^11^. Longitudinal studies of children also support a link between choroidal thickness changes and longer term eye growth in the human eye, with more rapid eye growth (i.e. during myopia development and progression) found to be associated with a thinning of the choroid, and slower axial eye growth associated with choroidal thickening in childhood^12,13^. Studies in humans examining short-term changes in choroidal thickness in response to a variety of factors have also shown that stimuli considered to be myopiagenic (e.g. accommodation^14^, hyperopic defocus^15,16^) are often associated with a transient thinning of the choroid and stimuli considered to be anti-myopiagenic (e.g. anticholinergic agents^17,18^, myopic defocus^16,19^) are associated with a transient choroidal thickening. These findings from both animal and human research suggest that choroidal thickness changes are one of the earliest observable ocular changes during the development of refractive errors, and that these changes may represent a biomarker for the signaling cascade that results in longer term changes in ocular growth and the development of refractive errors in response to environmental stimuli.

One environmental factor that has been implicated as playing a potentially important role in the regulation of eye growth and the development of myopia is ambient light exposure^20^. A number of epidemiological studies have demonstrated an association between increased outdoor activity and a lower risk of myopia development in children (see Xiong *et al*.^21^ for a review of these studies), which, given the high ambient light levels typically experienced outdoors compared to indoors, suggests that increased ambient light exposure may help to protect against myopia. Recent work using wearable sensors to objectively assess ambient light exposure in children has also shown an association between low levels of ambient light exposure and faster axial eye growth in children^22^. Although these findings support a potential influence of light exposure upon refractive error development, it is also possible that other factors such as altered retinal image blur associated with outdoor exposure may also play a role in the observed associations. An interventional study of indoor lighting in Chinese schools has demonstrated that increasing indoor illumination (by on average ~400 lux) in classrooms resulted in significantly lower myopia incidence and axial eye growth (and a significantly smaller change in refractive error in non-myopic, but not myopic children) compared to children attending control schools where lighting levels in classrooms were not increased^23^. This further supports a role for light exposure in the control of human eye growth, and suggests that relatively modest changes in ambient light levels have the potential to impact upon ocular growth. Consistent with ambient light levels influencing myopia development, bright light exposure has also been shown to protect against the development of form deprivation myopia in a range of animal species^20,24,25^.

Interestingly, studies with animals indicate that increased ambient light exposure can also result in choroidal thickening^26,27^, which suggests a potential involvement of the choroid in the mechanisms underlying the protective effects of ambient light exposure on myopia development. The effects of increased ambient light exposure upon choroidal thickness in the human eye however, have been less well studied. Light therapy (where a light emitting device is used to expose the eye to a controlled dosage of light at a specific time each day) is one method of increasing daily light exposure, and has been used previously for the treatment of circadian rhythm disturbances and seasonal affective disorder^28^. However, the ocular effects of light therapy have not been examined in detail previously. Therefore in this study, to better understand the impact of light exposure on the human choroid, we examined the effects of a 1-week period of light therapy (through the daily use of light therapy glasses worn for 30-minutes each morning) upon the choroidal thickness of a group of healthy young adult subjects with a range of refractive errors.

## Methods

Twenty three healthy young adult subjects aged between 20 to 29 years (mean ± SD age 22.2 ± 2.2 years) participated in this study and had a series of choroidal thickness measurements collected before, and then following a 1-week period of increased daily light exposure (induced through the daily use of light therapy glasses). The participants were either of Asian (61%) or Caucasian (39%) ethnic origin, and 65% were female. All subjects exhibited visual acuity with their best sphero-cylindrical refraction of logMAR 0.00 or better, and had a range of spherical equivalent refractive errors (SER) ranging from +1.00 to −5.75 D (mean ± SD SER −1.21 ± 2.15 D). No participants exhibited anisometropia of >1.00 D or astigmatism of >−1.50 DC (mean ± SD astigmatic refractive error −0.26 ± 0.44 DC). Any soft contact lens wearers were asked to abstain from lens wear 48 hours prior to ocular measurements in the study, and no rigid gas permeable contact lens wearers were included. All subjects were in good general health and had no history of ocular surgery or injuries. Subjects were screened prior to testing to exclude anyone with a condition known to influence circadian rhythms (e.g. insomnia, seasonal affective disorder, jet lag); or any optical or pharmacological myopia control treatments. Prior to commencing the study, approval from the Queensland University of Technology human research ethics committee was obtained, and all subjects provided written informed consent to participate and were treated in accordance with the tenets of the Declaration of Helsinki. The study procedures were carried out in accordance with the approved guidelines.

All participants in the study had a series of choroidal thickness measurements captured using spectral domain optical coherence tomography (SD-OCT) on two ocular measurement days that were conducted before (baseline measurement day) and then following 1-week of morning light therapy (post-light therapy measurement day). On each measurement day, 5 measurement sessions were conducted, approximately every 3 hours, with the first measurement session conducted at ~09:00 and the final measurement session at ~21:00 (with all measurements collected within ±60 minutes of the scheduled hour of testing, and 69% of measurements collected within ±30 minutes of the scheduled testing hour). All measurement sessions were conducted on weekdays. On each measurement day, participants were asked to abstain from consuming caffeinated or alcoholic beverages, to limit the potential confounding effects of alcohol^29^ and caffeine^30^ upon choroidal thickness. To reduce the influence of previous visual tasks (e.g. near work^14^) upon measurements, participants performed a 10-minute distance viewing task prior to each measurement session. Room illumination was kept at low photopic levels (~10 lux), during this distance viewing task and for all ocular measures. Ocular biometry measures were also collected at each session using the Lenstar LS 900 (Haag Streit AG, Koeniz, Switzerland) optical biometer, and these data were used to adjust the transverse scale of the OCT scans to account for ocular magnification effects due to differences in eye biometry. At each measurement session, subjects were also asked to report upon the time they spent performing near/intermediate tasks in the 3 hour period preceding each measurement.

**Figure 1:**
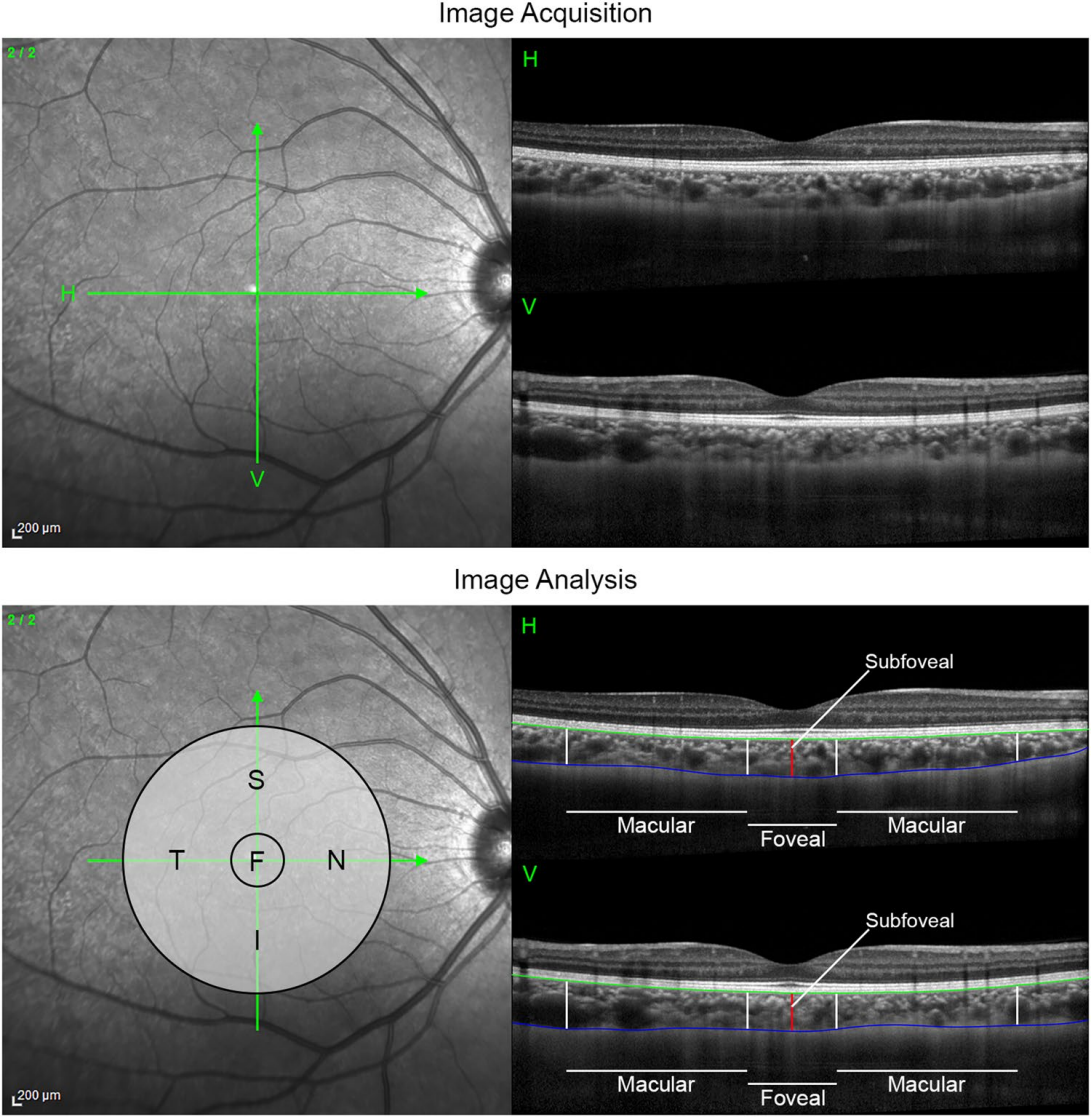
Illustration of the OCT scanning protocol and image analyses performed in the study. At each measurement session high resolution chorioretinal OCT images (each consisting of the average of 40 B-scans) were collected using a “cross” scanning protocol to capture 20° wide horizontal and vertical, foveal centred cross-sectional images (top panel). Each cross-sectional OCT image was analysed using semi-automated software to segment the retinal pigment epithelium (RPE, green line) and the chorioscleral interface (CSI, blue line) (bottom panel). These data were then used to calculate the subfoveal choroidal thickness; the foveal (F) region choroidal thickness (average thickness across the central 1 mm region); and the macular choroidal thickness (average thickness in a 2 mm region adjacent to the foveal region) in the superior (S), inferior (I), temporal (T) and nasal (N) regions.

Choroidal thickness measurements were derived from OCT images collected with the Heidelberg Spectralis OCT device (Heidelberg Engineering, Heidelberg, Germany). This instrument uses a super luminescent light source of central wavelength of 870 nm to provide cross sectional images of the posterior eye with a digital axial resolution of 3.9 µm. At each measurement session, two high-resolution “cross” scans were collected (each consisting of a vertical and a horizontal 20° long cross-sectional chorio-retinal image centered on the fovea) (Fig. 1). Each cross-sectional image was the average of 40 B-scans, and all measurements were collected using enhanced depth imaging mode^31^, to improve the visibility of the chorio-scleral interface. Only images with quality-index of >25 dB/50 dB were included (mean quality index 37.9 ± 2.5 dB). The instrument’s real-time eye tracking, follow-up imaging mode was used, in order to register all subsequent measurements to the same retinal location as the first measurement of the baseline measurement day.

On the day immediately following the baseline ocular measurements, each participant began a 7-day period of increased daily ocular light exposure, delivered through the use of commercially available light therapy glasses that were worn by each participant for 30 minutes each morning (Re-timer glasses, Re-Timer Pty Ltd, Australia). These light therapy glasses direct blue-green light (peak wavelength 500 nm) of illuminance 506 lux, towards the eye through 4 light emitting diodes positioned on the lower portion of the plastic frame ~20 mm from the eye. These light therapy glasses are recommended by the manufacturer for the treatment of sleep disturbances, jet lag and seasonal affective disorder, and have been shown to significantly alter circadian rhythms in healthy young adults^28^. Participants were instructed to wear the light therapy glasses for 30 minutes every morning at 7am (or when they woke up, if this was after 7am), while performing a distance visual task under indoor illumination and wearing their habitual distance refractive correction. Text messages were sent to participants each day, reminding them to wear the light therapy glasses, to encourage wearing compliance. Participants also completed a diary, in which they recorded the time each day when the light therapy glasses were worn. The second ocular measurement day (Post-light therapy measurement day) was conducted on the seventh day of light therapy treatment.

In order to examine each participant’s habitual daily light exposure and sleep patterns throughout the study, each subject wore a wrist-watch actigraphy device (Actiwatch 2, Phillips Respironics) on their non-dominant wrist 24-hours a day for a 14-day period, encompassing both the baseline measurement day and the post-light therapy measurement day. This device contains a silicon photodiode light sensor to measure white light illuminance (dynamic range, 5–100,000 lux, peak sensitivity 570 nm) and a solid state accelerometer for measurement of physical activity. All devices were programmed to collect instantaneous readings of light exposure and physical activity every 60 seconds. Participants were instructed to begin wearing the Actiwatch 7-days prior to their baseline measurement day, and to wear the watch continuously for 14-days, finishing wear after the final (post-light therapy) ocular measurement day.

### Data Analysis

The OCT images were exported from the instrument following each measurement session and were analysed with a semi-automated procedure using custom written software. Initially each OCT scan was analysed using an automated segmentation algorithm^32^ to segment the outer surface of the retinal pigment epithelium (RPE) and the chorio-scleral interface (CSI). An experienced, masked observer then checked the segmentation integrity of each scan and manually corrected any segmentation errors. The transverse scale of each scan was also corrected to account for ocular magnification effects based upon each subject’s ocular biometry measures collected at each measurement session. These corrected segmentation data were then used to calculate choroidal thickness (the axial distance from the RPE to the CSI) at the subfoveal point, the foveal region (the average thickness in the 1 mm wide region centred on the fovea), and in the superior, inferior, nasal and temporal macular region (the average thickness in the 2 mm wide region adjacent to the foveal region in each scan) (Fig. 1). One participant was excluded from analyses due to poor OCT image quality, and so the final analysis included data from 22 subjects.

The light exposure and physical activity data from the 14-day period of actigraphy were analysed using Actiware software version 6.0. Given that the vast majority of light exposure occurred between the hours of 5am and 9 pm across the measurements in the study, average hourly light exposure (between 5am and 9 pm) was derived from each subject’s Actiwatch data for the 7-day period before light therapy, and for the 7-day period of light therapy. Since the Actiwatch was worn on the wrist, the increased daily exposure associated with the light therapy glasses was not recorded by the device. Therefore, in order to provide an estimate of the increased light exposure during light therapy, an additional 500 lux was added to each ambient light exposure measurement during the 30-minute period of morning light therapy at the diary-reported daily wearing time for each subject (assuming an additive effect of the light therapy glasses with the ambient illumination). The average daily minutes of outdoor light exposure (daily time exposed to light levels >1000 lux) was also calculated from each subject’s Actiwatch data. The average daily bed time, wake time and sleep time was also calculated for these two seven day periods, using the automated sleep detection algorithms of the Actiware software (these algorithms analyse variations in activity data over the course of the day to determine participants sleep and awake status, and have previously been shown to provide reliable estimates of sleep patterns^33^). The datasets analysed in the current study are available from the corresponding author on reasonable request.

All statistical analyses were performed using IBM SPSS version 23.0 (IBM Corp, Armonk, NY, USA). The Kolmogorov-Smirnov test revealed that all parameters (with the exception of the hourly light exposure measurements) did not depart significantly from a normal distribution (p > 0.05). A log transformation of the daily hourly light exposure data was therefore performed prior to statistical analysis in order to correct the violation of the normality assumption. To examine the variations in subfoveal choroidal thickness associated with light therapy, a repeated measures analysis of variance (ANOVA) with two within-subject factors (time of day, and light exposure status) was employed. The macular choroidal thickness data were analysed using a similar approach, including an additional within-subject factor of choroidal region. A repeated measures ANOVA was also used to examine whether the average hourly light exposure varied significantly over the course of the day, and during the 1-week period of light therapy. Paired t-tests were used to assess whether significant change occurred in the minutes of daily outdoor light exposure and sleep patterns following the 1-week period of light therapy. The repeatability of the choroidal thickness estimates were assessed using the methods of Bland and Altman^34^ to determine the mean difference and 95% limits of agreement (LOA) between the 2 repeated measures of choroidal thickness collected at each measurement session.

## Results

### Repeatability of choroidal thickness measures

Analyses revealed a high level of repeatability for the measures of choroidal thickness in each region, with negligible difference between repeated measures and narrow limits of agreement, consistent with previous studies of choroidal thickness repeatability^35^. For the subfoveal thickness measures, the mean ± SD difference between the two repeated measures at each measurement session was +0.2 ± 8.7 µm (95% LOA: +17.3 to −16.8 µm). Similar results were found for the macular choroidal thickness estimates, in the foveal region (mean difference: +0.3 ± 7.8 µm, 95% LOA: +15.6 to −15.0 µm), superior macular region (−0.3 ± 10.3 µm, +19.9 to −20.5 µm), nasal macular region (+0.1 ± 8.2 µm, +16.2 to −16.1 µm), inferior macular region (+0.4 ± 8.2 µm, +16.6 to −15.7 µm) and temporal macular region (−0.2 ± 7.4 µm, +14.3 to −14.8 µm).

### Morning light therapy and subfoveal choroidal thickness variations

**Figure 2:**
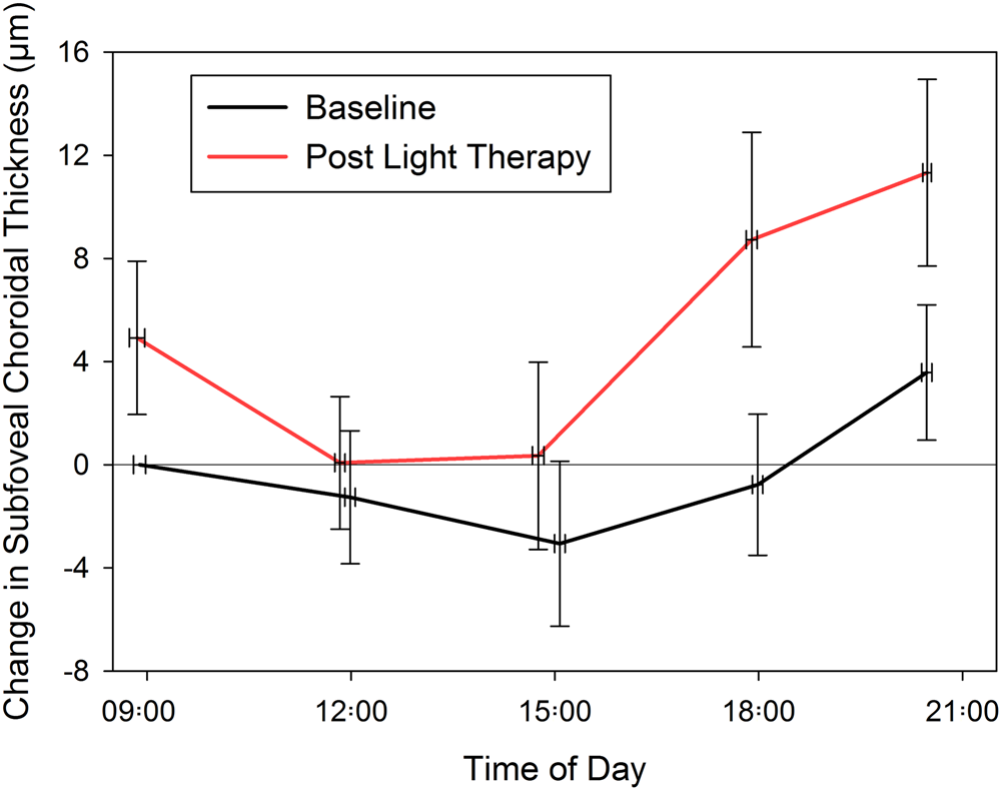
Mean daily change in subfoveal choroidal thickness at baseline and following 7 days of morning light therapy. All changes are normalized to the first measurement on the baseline measurement day. Vertical error bars represent ± standard error of the mean change in choroidal thickness and horizontal error bars are the standard error of the mean measurement time.

Table 1 and Fig. 2 illustrate the mean choroidal thickness and their daily variations at baseline and following a 1-week period of morning light therapy. Repeated measures ANOVA revealed that subfoveal choroidal thickness varied significantly with time of the day (p < 0.001), indicative of significant diurnal variations in choroidal thickness over the course of the study. The subfoveal choroidal thickness was typically observed to be at a minimum during the day and to show a thickening in the evening. Bonferroni adjusted pairwise comparisons revealed the choroidal thickness at measurement session 2 (mean time 11:55 ± 0:22) and session 3 (mean time 14:55 ± 0:25) was significantly thinner than the choroidal thickness at measurement session 5 (mean time 20:29 ± 0:29) (all p < 0.01).

**Table 1:** Mean ± SD choroidal thickness and daily amplitude of change (difference between the maximum and minimum choroidal thickness measurement over the course of the measurement day) in choroidal thickness and their changes following a 1-week period of morning light therapy.

	Choroidal thickness (µm)		Daily amplitude of change (µm)	
	Mean at baseline	Change post light therapy	Mean at baseline	Change post light therapy
Subfoveal Point	345.4 ± 88.3	+5.4 ± 10.3	14.6 ± 10.0	+5.1 ± 14.7
Foveal Region	344.1 ± 86.9	+5.4 ± 9.8	14.3 ± 8.3	+5.3 ± 13.3
Superior Macula	352.2 ± 73.8	+4.9 ± 8.8	19.2 ± 11.8	+7.3 ± 15.5
Nasal Macula	273.6 ± 76.4	+3.6 ± 6.3	12.7 ± 7.8	+5.1 ± 12.6
Inferior Macula	344.9 ± 80.6	+4.0 ± 8.1	14.2 ± 6.7	+3.8 ± 10.9
Temporal Macula	326.1 ± 82.0	+3.2 ± 7.9	12.9 ± 7.3	+2.9 ± 11.4

A significant effect of light therapy upon subfoveal choroidal thickness was also found, with a 1-week period of morning light therapy found to result in a small but statistically significant increase in choroidal thickness (mean increase +5.4 ± 10.3 µm; 95% CI +0.8 to +9.9 µm; p = 0.02). Although Fig. 2 demonstrates a trend for the increase in choroidal thickness associated with light therapy to be more prominent in the evening (measurement sessions 4 and 5), there was no significant time of day by light exposure interaction (p = 0.1), indicating that the slight thickening of the choroid following light therapy was relatively constant across the day. The average daily amplitude of subfoveal choroidal thickness variation (i.e. the difference between each subject’s maximum and minimum choroidal thickness measure each day) exhibited a small magnitude increase following light therapy (mean increase +5.1 ± 14.7 µm; 95% CI −1.4 to 11.6 µm), however this change did not reach statistical significance (p = 0.1).

### Morning light therapy and macular choroidal thickness variations

**Figure 3:**
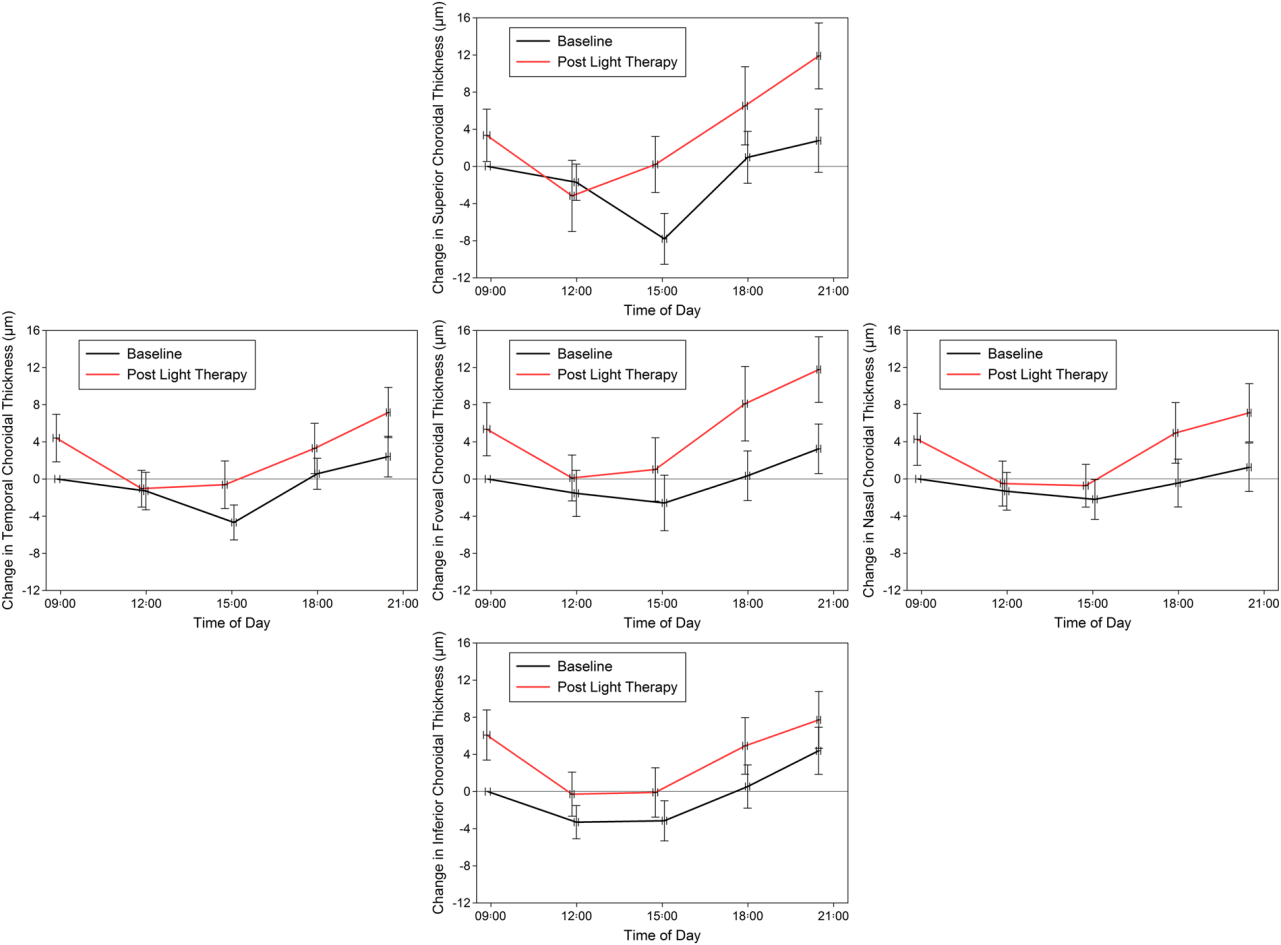
Mean daily change in choroidal thickness across the macular region at baseline and following 7 days of morning light therapy. The mean choroidal thickness changes in the foveal region (centre), superior macula region (top), inferior macular region (bottom), nasal macular region (right) and temporal macular region (left) are illustrated. All changes are normalized to the first measurement on the baseline measurement day. Vertical error bars represent ± standard error of the mean change in choroidal thickness and horizontal error bars are the standard error of the mean measurement time.

The choroidal thickness and its changes following light therapy in each of the considered macular regions are illustrated in Fig. 3 and Table 1. A significant overall difference in thickness across the macular region was found (p < 0.001), with the thickness of the nasal region found to be significantly thinner than all other considered regions. Similar to the variations in the subfoveal choroid, a significant effect of time of day (p < 0.001) was observed in the macular choroid, with choroidal thickness at measurement sessions 2 and 3 again being significantly thinner than measurement session 5 (all p < 0.01). Light therapy was also found to result in a significant increase in the average macular choroidal thickness (mean increase +4.7 ± 8.2 µm; 95% CI +0.9 to +7.5 µm; p = 0.01). There were no significant light by time, light by region or light by time by region interactions observed (each p > 0.05), indicating that the increase in choroidal thickness following light therapy did not appear to be significantly different according to the time of the day or the choroidal region assessed. For the macular choroid, the daily amplitude of thickness change showed a small increase following light therapy (mean increase +5.1 ± 11.7 µm; 95% CI +0.02 to +9.8 µm), which just reached statistical significance (p = 0.049). A significant effect of region was also observed for the daily amplitude of change (p < 0.001), with the superior macular region exhibiting a significantly higher amplitude of change compared to the inferior, nasal and temporal macular regions. The amplitude of change did not however exhibit a significant region by light exposure interaction, indicating that the increase in amplitude following light therapy did not differ significantly between regions (p = 0.33).

### Daily ambient light exposure and sleep patterns

**Figure 4:**
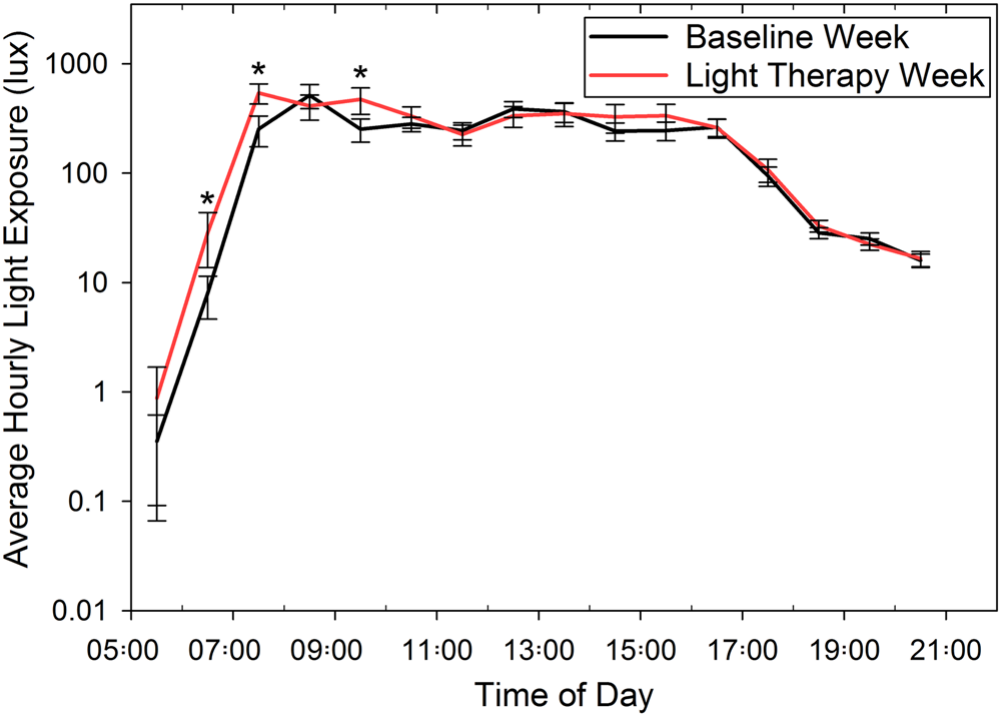
Average hourly light exposure for the 7-day period prior to the baseline measurements (black line), and during the 7-day period of morning light therapy (red line). Asterisks indicate periods of time where the average hourly light exposure was significantly greater (p < 0.05) during the week of morning light therapy. Error bars represent ± the standard error of the mean hourly light exposure. The extra light exposure due to the light therapy was accounted for in this analysis by adding an additional 500 lux of light exposure to the recorded ambient light exposure for each minute of the diary-reported 30-minute period that each participant wore the light therapy glasses on each day of the light therapy week (the mean reported time that the light therapy glasses were worn was 07:08 am ± 00:51 min; range: 05:30 to 11:01am).

Figure 4 illustrates the average hourly light exposure between 5am and 9 pm assessed with data from the wrist worn light sensors worn by each subject for the 7-day period prior to the baseline measurements and for the 7-day period of light therapy (including the additional 500 lux of light exposure for each minute of the diary-reported 30-minute period that each participant wore the light therapy glasses on each day of the light therapy week). The average reported time that the light therapy glasses were worn was 07:08 am ± 00:51 min (range: 05:30 to 11:01am). Repeated measures ANOVA revealed a significant effect of light therapy (p = 0.02), indicative of the average daily light exposure between 5am and 9 pm over the 7-days of light therapy (mean 431 ± 397 lux) being significantly greater than during the baseline week (mean 314 ± 193 lux). A significant light therapy by time interaction was also observed (p = 0.01), with significantly greater average hourly light exposure during the 7-day light therapy period, observed in the morning between 6am to 8am, and 9am to 10am (all p < 0.05), but no significant differences in hourly light exposure between the baseline and light therapy periods at any other times of the day. The average daily wake time was found to be significantly earlier (mean difference 14 ± 25 minutes; 95% CI: 3 to 26 minutes) during the light-therapy period (p = 0.02) compared to the baseline period, however the bed time and average sleep time each night were not significantly different between the two measurement periods (both p > 0.05) (Table 2). There was no significant difference in the average daily minutes of bright outdoor light exposure (>1000 lux) associated with light therapy (mean difference +5 ± 22 minutes; 95% CI: +15 to −5 minutes; p = 0.33). The amount of time that subjects reported spending on near/intermediate tasks did not differ significantly between the baseline and post light therapy measurement days (mean difference of 21 ± 275 minutes; 95% CI: −140 to +98 minutes; p = 0.7).

**Table 2:** Mean ± SD daily outdoor (>1000 lux) light exposure and sleep/wake times derived from 7-days of actigraphy measurements collected prior to the baseline measurement day and during the 1-week period of daily light therapy.

	Daily minutes of outdoor light (>1000 lux) exposure	Bed time (24 hour time)	Wake time (24 hour time)	Sleep time (hours:minutes)
Baseline week	43 ± 26	23:08 ± 1:29	07:31 ± 0:54	8:25 ± 1:09
Light therapy week	48 ± 40	23:05 ± 1:17	07:17 ± 0:56	8:15 ± 0:49
*p-value*	0.33	0.81	0.02	0.27

P-values represent the outcome from a paired t-test comparing the baseline week and the light therapy week.

## Discussion

This study provides the first evidence in the human eye, that increasing daily light exposure through 30-minute light therapy sessions in the morning for a 7-day period, results in a small magnitude but statistically significant increase in choroidal thickness. The choroidal changes observed appeared to be characterised by a small overall increase in choroidal thickness, coupled with evidence for a small increase in the diurnal amplitude of change, but no apparent significant change in the phase of the diurnal variations in choroidal thickness. Although the magnitude of choroidal change would not be considered clinically significant, these findings add to our knowledge of environmental factors that can influence the choroid in the human eye, and given the potential importance of both light exposure^20–25^ and the choroid^1,5,12^ in the regulation of eye growth, may have implications for our understanding of environmental factors contributing towards refractive error development in the human eye.

Our average macular choroidal thickness measures in young adults on the baseline day prior to light therapy are consistent with previous studies of choroidal thickness in healthy human eyes that also report a significantly thinner choroid in the nasal macular regions approaching the optic nerve head^11,36^. The pattern of diurnal choroidal thickness observed are comparable (although slightly smaller on average) in amplitude, and of similar phase to a number of previous studies of choroidal diurnal rhythms in normal human subjects, that have noted daily amplitudes of change ranging from 20 µm to 60 µm microns and also typically find the thinnest choroid to occur during the day at midday or early afternoon^7,9,10^. We also found some regional variations in the magnitude of diurnal change in choroidal thickness, with the superior macular region exhibiting the highest amplitude of diurnal change. This finding is consistent with Gabriel *et al*.^37^ who reported the highest magnitude of choroidal diurnal variation (between morning and early evening) occurred in the superior outer macula region.

Results from both animal^1–4^ and human^11^ studies suggest that choroidal thickness changes occur early in the process of refractive error development and appear be associated with longer term changes in ocular growth. If short-term choroidal thickness changes are considered as a biomarker of longer term ocular growth, then we speculate that the findings from our current study of choroidal thickening associated with light therapy, suggest that increasing ambient light exposure could potentially slow eye growth in the longer term. These findings also support the potential involvement of the choroid in the mechanisms linking ambient light exposure with alterations in ocular growth. Further research examining the ocular changes in response to increased light exposure over a longer period of time however, is required to definitively establish a link between increasing daily light exposure and eye growth and refractive development in the human eye.

Previous studies of both avian^26^ and mammalian^27^ animal models have also demonstrated that exposure to bright light during the day can result in an increase in choroidal thickness. Although these animal studies differ from our current study in terms of experimental protocols and the magnitude of choroidal thickness change observed, our findings of a significant increase in choroidal thickness following light therapy are broadly consistent with these previous findings. Interestingly, Lan and colleagues^26^ examined the effects of a 6-hour period of exposure to bright light during the day on the chick choroid, and reported a significant thickening of the choroid in the 4-hour period of the afternoon/evening following the bright light exposure. This is also consistent with our findings, where an increase in choroidal thickness was observed following light therapy at 7am, with the largest magnitude increases observed in the afternoon/evening, some hours after the initial light therapy exposure.

Although our study is the first to examine changes in human choroidal thickness following morning light therapy, previous studies of human eyes have reported on a short-term increase in choroidal blood flow when subjects transition from dark to light conditions^38^. A recent study of healthy adults also reported upon choroidal thickness changes in response to increased light exposure at night. In contrast to our finding of choroidal thickening in response to increased morning light exposure, Ahn *et al*.^39^ reported that a 4-hour period of increased light exposure at night, immediately before sleep, resulted in a significant thinning of the choroid. A study with the chick animal model also demonstrated that 2 hours of light exposure in the middle of the night resulted in a disruption to the normal sinusoidal diurnal rhythm in choroidal thickness and an increase in ocular growth rate compared to control animals reared under a normal light/dark cycle^40^. These contrasting findings between our current study examining morning light therapy and studies of increased light exposure at night, suggest that the response of the human choroid to increased illumination, may be dependent upon the time of day of light exposure, which suggests a potential complex interaction between the eye’s normal diurnal rhythms and the influence of environmental factors such as light exposure upon the choroid. Recent animal studies also indicate that the ocular response in the chick to environmental optical stimuli (myopic and hyperopic defocus and form deprivation) appear to differ depending on the time of day of exposure, suggesting that the mechanisms regulating eye growth may exhibit diurnal rhythms in their sensitivity and appear to be highly dependent upon the time of day^41,42^.

Our experimental protocol involved 30-minutes of ocular exposure to blue-green light in the morning each day. Since this intervention involved exposure to increased illuminance and to specific wavelengths of light, the relative importance of light intensity and spectral content in eliciting the observed choroidal responses is not clear. It is also worth noting that the light therapy method employed, involved a relatively modest increase in illuminance for a relatively brief period each day (~500 lux for a 30 minute period). Increasing daily outdoor activity, an intervention that has been shown to protect against myopia development in childhood^43^, would be expected to involve exposure to substantially higher intensity light, of different spectral properties compared to our current study. Further research is required to examine the effects of increased light intensity and spectral content as well as the duration and timing of exposure, upon the choroidal changes occurring following increased daily light exposure.

Although the mechanism linking daily light exposure with an increase in choroidal thickness is not clear from our current study, Lan *et al*.^26^ proposed a potential mechanism involving a light induced increase in retinal dopamine resulting in nitric oxide release and subsequent thickening of the choroid, underlying the choroidal thickening following light exposure in the chick eye. This mechanism seems plausible in the human eye also, given the known effects of increased light exposure upon retinal dopamine^44^ and the role of nitric oxide in choroidal blood flow changes in response to changing light conditions^45^. The short wavelength blue-green light used by the light therapy glasses in this study also supports a potential involvement of melanopsin signaling in the choroidal changes observed, since intrinsically photosensitive retinal ganglion cells have their peak sensitivity to short wavelength light^46^. Involvement of melanopsin signaling in light induced choroidal thickening is supported by Berkowitz and colleagues^27^ who found that mice lacking melanopsin photopigment did not exhibit choroidal thickening during transition from dark to light conditions.

Light therapy is also known to influence circadian rhythms^28^, and the significant changes observed in the wake time following light therapy suggests that some changes in our participants’ circadian rhythms occurred as a result of the morning light therapy in our current study. It is therefore possible that changes in circadian rhythms in the choroid as a result of morning light therapy may have contributed towards some of the observed changes in choroidal thickness in our current study. The close link between ambient light exposure and circadian rhythms makes it difficult to disentangle the relative contribution of choroidal thickening as a direct response to increased light exposure and changes occurring indirectly as a result of altered circadian rhythms, to the changes observed in choroidal thickness in our current study. Further research is therefore required to better understand the relative contribution of these two factors, and their interactions in the choroidal changes occurring as a result of light therapy.

A limitation of our current study design is that participants performed their normal everyday activities on each of the measurement days, leading to the possibility that between day differences in environmental exposures could influence the findings. However, actigraphy measurements indicated that apart from a significant increase in light exposure in the morning hours during the light therapy week, the average hourly light exposure did not differ significantly between days, suggesting that apart from the increase in light exposure associated with the morning light therapy, the ambient light exposure was otherwise similar between the two measurement days. Similarly, the daily hours of near work reported by subjects did not differ between baseline and light therapy measurement days, suggesting similar visual demand between the two ocular measurement days.

In our experimental protocol, participants wore the light therapy glasses for a 30-minute period in the morning, while they performed a distance viewing task. Since near focusing has been shown to lead to a small magnitude but statistically significant choroidal thinning^14,47^, this leaves open the possibility that changes in accommodative tone during the delivery of light therapy may have contributed towards the changes in choroidal thickness observed. However, choroidal thickness changes associated with accommodation are known to be transient (i.e. dissipate within 10 minutes of a near task), and are only significant for relatively high accommodation demands (>3D). This suggests that it is unlikely that the 30 minute distance task in the morning would have substantially influenced the choroidal measurements in our study.

## Conclusions

This study furthers our knowledge of environmental factors that can influence the human choroid, by demonstrating that morning light therapy for a 7-day period results in a small but statistically significant increase in choroidal thickness. Since there is evidence of a link between short term choroidal thickness changes and longer term ocular growth changes, these findings may have implications for our understanding of the impact of increased daily light exposure upon refractive error development. However, further longitudinal research examining children with progressing myopia is required to more clearly understand the longer term effects of increased daily light exposure and the impact of time of day of light exposure (which may influence and interact with circadian rhythms) on eye growth regulation and refractive error.
